# Mesenchymal Stem Cell Graft Improves Recovery after Spinal Cord Injury in Adult Rats through Neurotrophic and Pro-Angiogenic Actions

**DOI:** 10.1371/journal.pone.0039500

**Published:** 2012-06-20

**Authors:** Renaud Quertainmont, Dorothée Cantinieaux, Olivier Botman, Selim Sid, Jean Schoenen, Rachelle Franzen

**Affiliations:** GIGA Neurosciences, Axonal Regeneration and Cephalic Pain Unit, University of Liege, Liege, Belgium; University of Medicine and Dentistry of New Jersey, United States of America

## Abstract

Numerous strategies have been managed to improve functional recovery after spinal cord injury (SCI) but an optimal strategy doesn't exist yet. Actually, it is the complexity of the injured spinal cord pathophysiology that begets the multifactorial approaches assessed to favour tissue protection, axonal regrowth and functional recovery. In this context, it appears that mesenchymal stem cells (MSCs) could take an interesting part. The aim of this study is to graft MSCs after a spinal cord compression injury in adult rat to assess their effect on functional recovery and to highlight their mechanisms of action. We found that in intravenously grafted animals, MSCs induce, as early as 1 week after the graft, an improvement of their open field and grid navigation scores compared to control animals. At the histological analysis of their dissected spinal cord, no MSCs were found within the host despite their BrdU labelling performed before the graft, whatever the delay observed: 7, 14 or 21 days. However, a cytokine array performed on spinal cord extracts 3 days after MSC graft reveals a significant increase of NGF expression in the injured tissue. Also, a significant tissue sparing effect of MSC graft was observed. Finally, we also show that MSCs promote vascularisation, as the density of blood vessels within the lesioned area was higher in grafted rats. In conclusion, we bring here some new evidences that MSCs most likely act throughout their secretions and not via their own integration/differentiation within the host tissue.

## Introduction

Treatment of spinal cord injury (SCI) faces several problems. First of all, the mechanical damage and axonal disruption in the spinal cord are followed by a progressive cascade of secondary deleterious reactions spreading to the adjacent spared tissue leading to lesion extension and worsening the situation [1]. Secondly, although axonal regeneration is initiated, it is quickly repressed due to the inhibitory environment acting as a chemical and physical barrier for repair [2]. In this context, the self-regeneration and reorganization ability of the central nervous system is insufficient to lead to considerable functional improvements.

Numerous strategies have been managed to improve functional recovery after SCI. These studies focused on neuroprotection or axonal regeneration, by modifying the injured environment to be beneficial for repair, by replacing lost cells, stimulating and guiding axonal growth or boosting remyelination [3], [4], [5], [6]. To act on these events, scientists often exploited the potential of cell therapy using transplantation of various cell types like Schwann cells [7], [8], olfactory ensheathing cells [9], [10], neural stem cells [11], [12], bone marrow stromal cells [13], [14], fibroblasts [15], [16] and macrophages [17]. Despite promising results, it ensues from these experiments and from the complexity of SCI that an optimal single focalized strategy to treat it efficiently doesn't exist. It becomes evident that research has to concentrate its effort on multifactorial treatments, playing on the different factors of SCI pathophysiology. In this context, it appears that bone marrow stromal cells, also called mesenchymal stem cells (MSCs) could take an interesting part in these strategies.

Indeed, MSCs are adult stem cells with self-renewing and differentiation abilities [18]. These cells are harvested from bone marrow and easily and quickly expanded *in vitro*. This accessibility makes them an interesting candidate for autologous transplantation, abolishing immune and ethical problems of embryonic/foetal stem cell grafts [19]. MSCs are also able to modulate the CNS injured environment and to promote repair as they secrete anti-inflammatory, anti-apoptotic molecules and trophic factors able to support axonal growth, immunomodulation, to promote angiogenesis, remyelination and to protect from apoptotic cell death [20], [21], [22], [23]. In addition, some evidence suggests that MSCs may be able to differentiate into neuronal cells *in vitro* using co-culture [24], [25] or differentiation medium [26], [27], [28] but also *in vivo* after transplantation [13], [29] making them a good candidate for neuronal cell replacement strategies.

Different modes of administration have been used for cell transplantation after SCI: intrathecal [30], intravenous [13] or intraspinal [31]. For a human application, intravenous injection appears to be the easiest way to bring therapeutics to patient without risking further damage to the spinal cord. Furthermore, MSC intravenous injection is safe, as it has been shown to have no adverse effect like obstruction of blood circulation or tumorigenicity [32], [33].

The aim of this study was to graft MSCs by intravenous injection one week after a spinal cord compression injury, in order to assess their effect on functional recovery and find by which mechanisms these cells exert their beneficial effect.

## Results

### 1. Characterisation of rat MSCs *in vitro*

**Figure 1:**
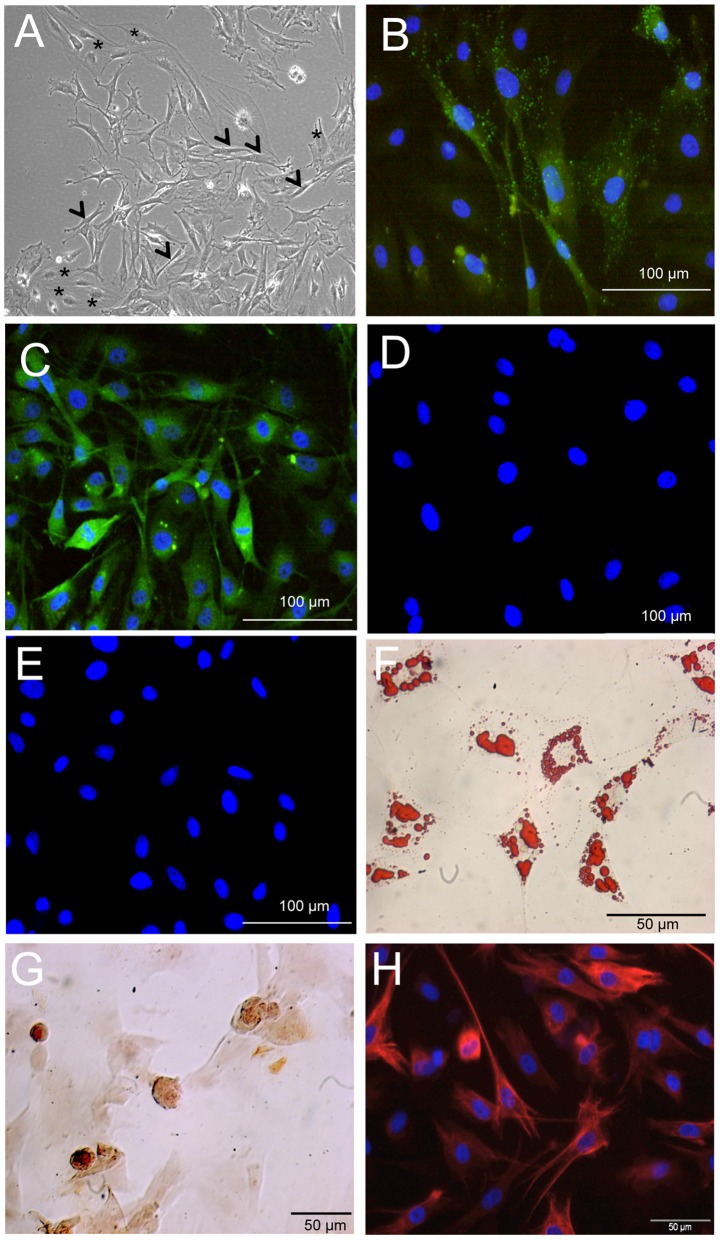
Mesenchymal stem cell characterization. (**A**): Phase contrast microphotography of rat MSCs in culture. Cells exhibit typical elongated, fibroblast-like morphology (arrowheads) or large, flattened shape (asterisks). (**B**) CD90 and (**C**) CD271 positive immunofluorescent labelings of P4 MSCs in culture, while CD45 **(D)** and CD11b (**E**) immunostainings are negative. (**F**) Adipogenic differentiation of P4-MSCs revealed with Oil Red O. (**G**) Alizarin Red staining of P4 MSCs induced to osteocytes reveals mineral deposition. (**H**) Immunofluorescent visualization of nestin induction in P12-MSCs deprived of serum. Nuclei are labeled with the Vectashield-DAPI mounting medium.

MSCs were isolated from Wistar adult female rats and cultured in plastic adherent conditions, classically used for MSCs *in vitro* expansion. They were characterized according to three criteria: adherence to tissue culture plastic dish, specific surface antigen expression and multipotent differentiation ability. MSCs exhibited typical elongated, fibroblast-like morphology or large, flattened shape (figure 1A). After 4–5 passages (P4–P5), all cells express surface antigens CD90 (figure 1B) as well as the neurotrophin co-receptor p75NGFr, also known as CD271 (figure 1C) [34], while they were negative for CD45 and CD11b (figures 1D and 1E). P4 MSCs are multipotent, as they differentiate into adipocytes and osteocytes according to published protocols (figures 1F and 1G) [35]. After 12 passages, cells were deprived of serum to induce the expression of nestin (figures 1H), a protein expressed by neural stem cells. This strategy was used in order to influence their fate towards a neural phenotype. Indeed, it has been demonstrated that only nestin-positive MSCs were able to differentiate into functional neurons *in vitro* [24]. The transcriptomic and proteomic comparisons of nestin-positive and nestin-negative MSCs also suggest that the nestin-positive cell population contains a higher percentage of stem cells that would adopt a neural fate [36]. It thus seemed appropriate to use these nestin-positive cells in our transplantation strategy.

### 2. BrdU labelling of MSCs

**Figure 2:**
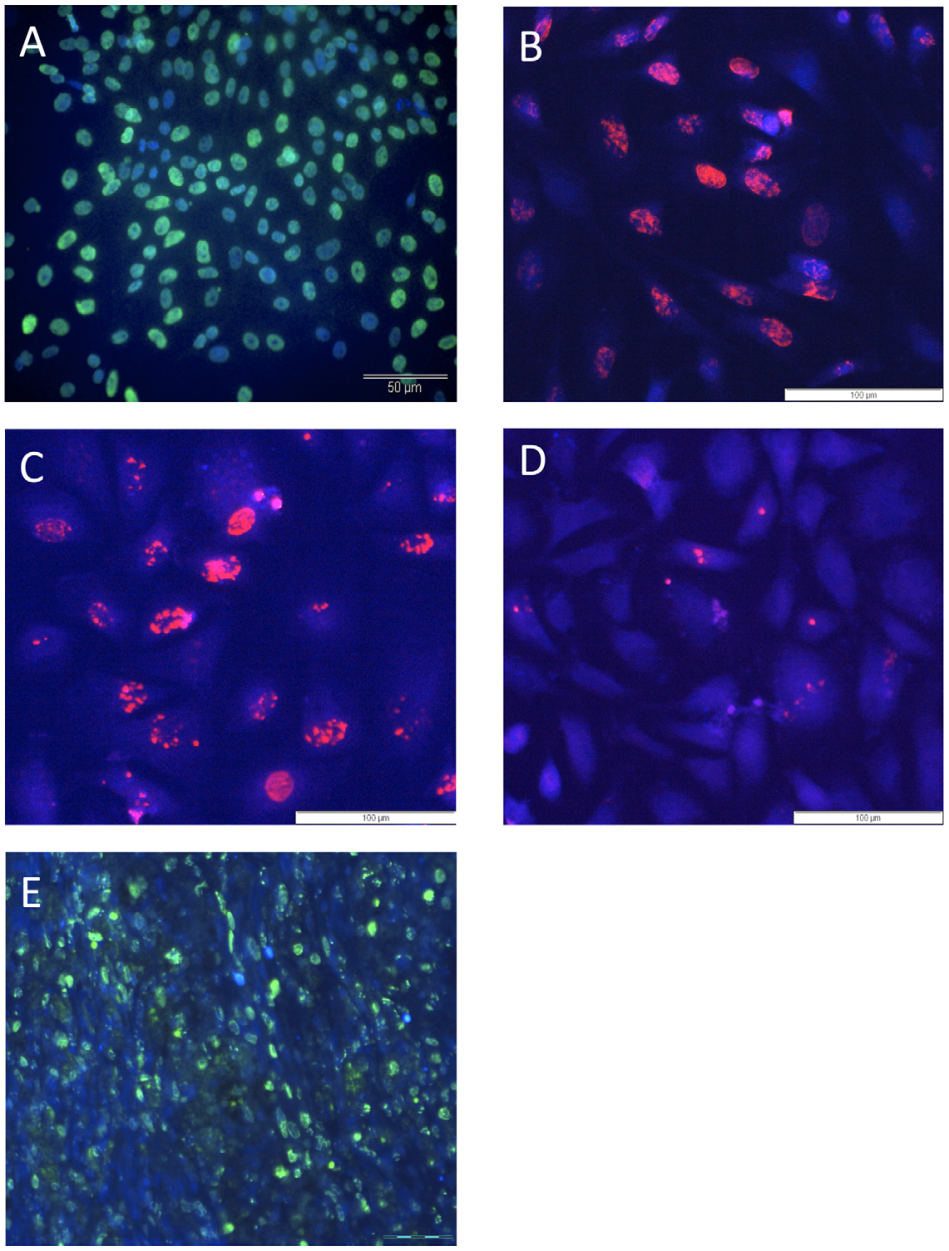
BrdU immunodetection. (**A**) BrdU (FITC)-DAPI immunostaining on MSCs cultured with 1.10^−6^M BrdU for 72 h, demonstrating that cells were all labeled before being transplanted. (**B–C–D**) BrdU (Rhodamine) maintenance in MSCs 3 days (**B**), 7 days **(C)** and 21 days (**D**) after the removal of BrdU from the culture medium. (**E**) BrdU immunodetection (FITC) on a longitudinal spinal cord tissue section from a rat that received 3 ip BrdU injections after spinal cord injury. Scale bar: 50 µm (A), 100 µm (B, C, D) and 200 µm (E).

We used *in vitro* BrdU incorporation to label MSCs before graft. After 72 h of culture in presence of 1.10^−6^M BrdU, we confirmed by immunocytochemistry that all MSCs were labelled with BrdU (figure 2A). We also assessed the maintenance of the BrdU labelling in MSCs with time to take into account cell population doublings and cell passages. We show that 3 and 7 days after the removal of BrdU from the culture medium, which respectively correspond to 0 and 1 passage, BrdU is still easily detected within the MSCs (figures 2B–C). After 21 days, and 6 passages, the signal is clearly reduced, but remains observable (figure 2D).

### 3. Fate of grafted cells

To study the fate of grafted cells, we performed, at 7, 14 and 21 days after the graft, a BrdU immunohistochemistry on the injured transplanted spinal cord, but also on lung, liver, spleen and kidney tissue sections, as MSCs were grafted in the tail vein. Unfortunately, no grafted cell could be found within the lesion site neither in any organs (data not shown). In order to rule out a problem of BrdU detection, we also performed BrdU immunohistochemistry on spinal cord sections from rats that received, during 3 consecutive days following SCI, intraperitoneal BrdU injection (100 mg/kg), and were allowed to survive for 2 more weeks. Figure 2E shows that BrdU is detected within the injured spinal cord, demonstrating that our protocol of detection is efficient.

### 4. Control of MSC viability and CD profile

**Figure 3:**
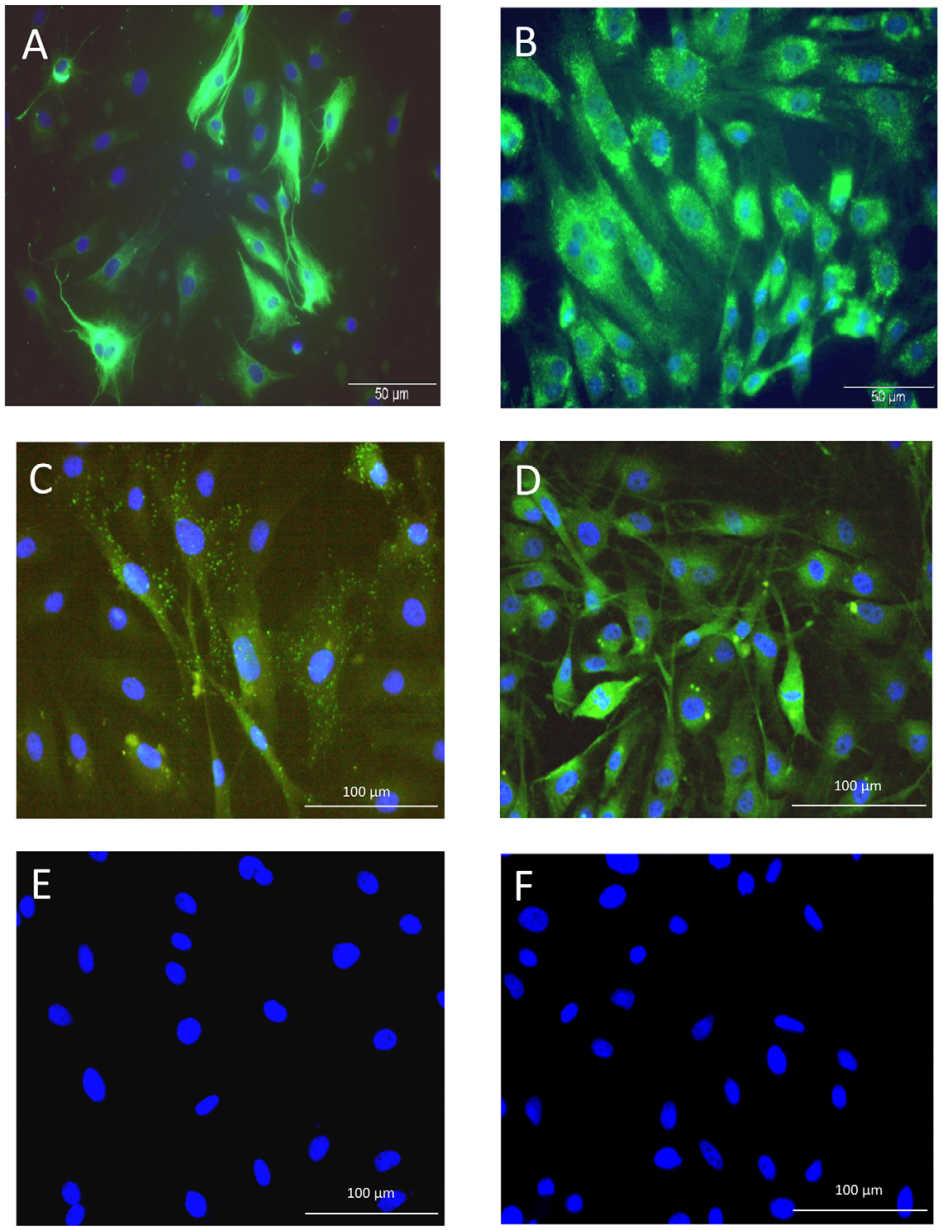
MSC viability and CD profile. Nestin (**A**) and p75NGFr (**B**) immunofluorescent stainings on MSCs: The last drop of MSC suspension from the injection needle was placed back into culture to check their viability at the time of injection. (**C**–**F**): CD profile of P12 MSC grafted cells, showing their immunoreactivity for CD90 (C) and CD271 (D), and the absence of CD45 (E) and CD11b (F) expression. Scale bar: 50 µm (A, B), 100 µm (C–F).

To confirm cell viability at the time of iv injection, we placed back into culture the last drop of the MSC suspension from the injection needle and characterized them 24 hours later by immunocytochemistry for nestin and p75NGFr. Transplanted cells were viable at the time of injection as they still could adhere to plastic culture flask and express nestin and p75NGFr (figures 3 A–B). As transplanted cells were P12 MSCs, we checked that their CD profile was still the one that characterizes MSCs, and thus performed CD90, CD271, CD45 and CD11b immunocytochemistry on P12 MSCs (figures 3 C–F). The results confirm that P12 MSCs have conserved their CD profile, as they do still express CD90 and CD271, and are still negative for CD45 and CD11b.

### 5. MSC graft improves functional recovery after spinal cord compression injury

**Figure 4:**
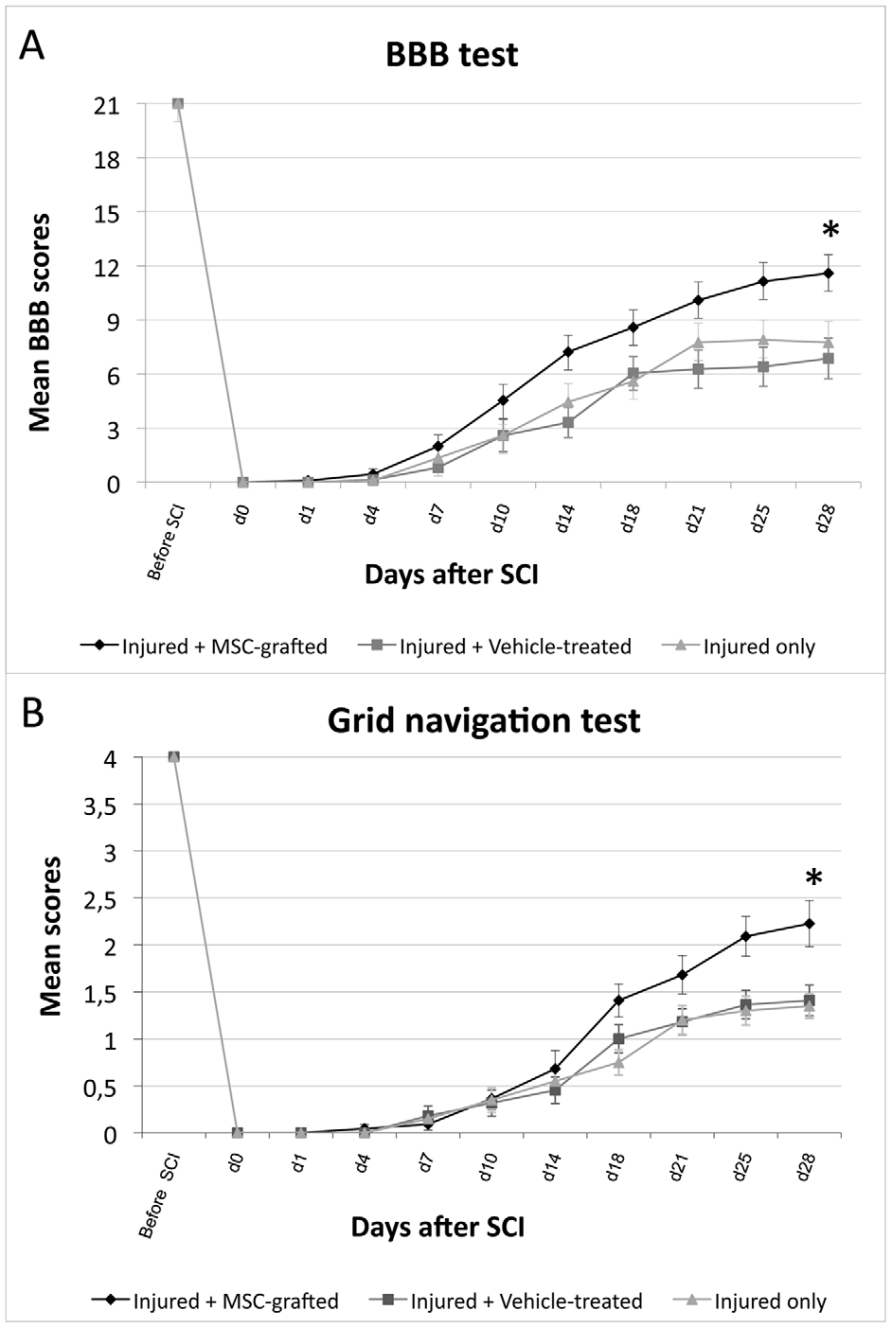
Behavioral analysis. (**A**) Locomotor scores as assessed by the BBB rating scale. MSC grafted rats reach significantly higher scores compared to both control groups. Only MSC transplanted rats reach the weight-supporting step level (score of 9). Injured + MSC vs injured only p = 0.0029; injured + MSC vs injured + vehicle p<0.0001. (**B**) Grid test scores assessing deficits in descending fine motor control. MSC grafted rats reach higher scores, significantly different from control rats. Injured + MSC vs injured only p<0.0001; injured + MSC vs injured + vehicle p<0.0001.

Spinal cord injured animals were submitted to 2 behavioural assessments in order to evaluate the effect of MSC graft on functional recovery. Locomotor skills were assessed by the open field test using the BBB rating scale. Mean scores over time were higher in the MSC grafted group (injured + MSC) compared to control groups (injured + vehicle or injured only). The difference between MSC grafted and control groups was statistically significant (injured + MSC vs injured only p = 0.0029; injured + MSC vs injured + vehicle p<0.0001; figure 4A). Moreover, only MSC grafted animals reached and overtook the score corresponding to weight support stepping (score of 9). Interestingly, locomotor scores from MSC grafted rats begun to be distinguishable from control groups already three days after the graft. The grid navigation test completes this behavioural part of motor recovery assessment. Even if grafted rats did not recover their toe control and the capacity to realise fine movements, their scores were significantly improved with MSC graft (injured + MSC vs injured only p<0.0001; injured + MSC vs injured + vehicle p<0.0001; figure 4B).

### 6. MSC graft increases NGF level expression within the injured tissue

**Figure 5:**
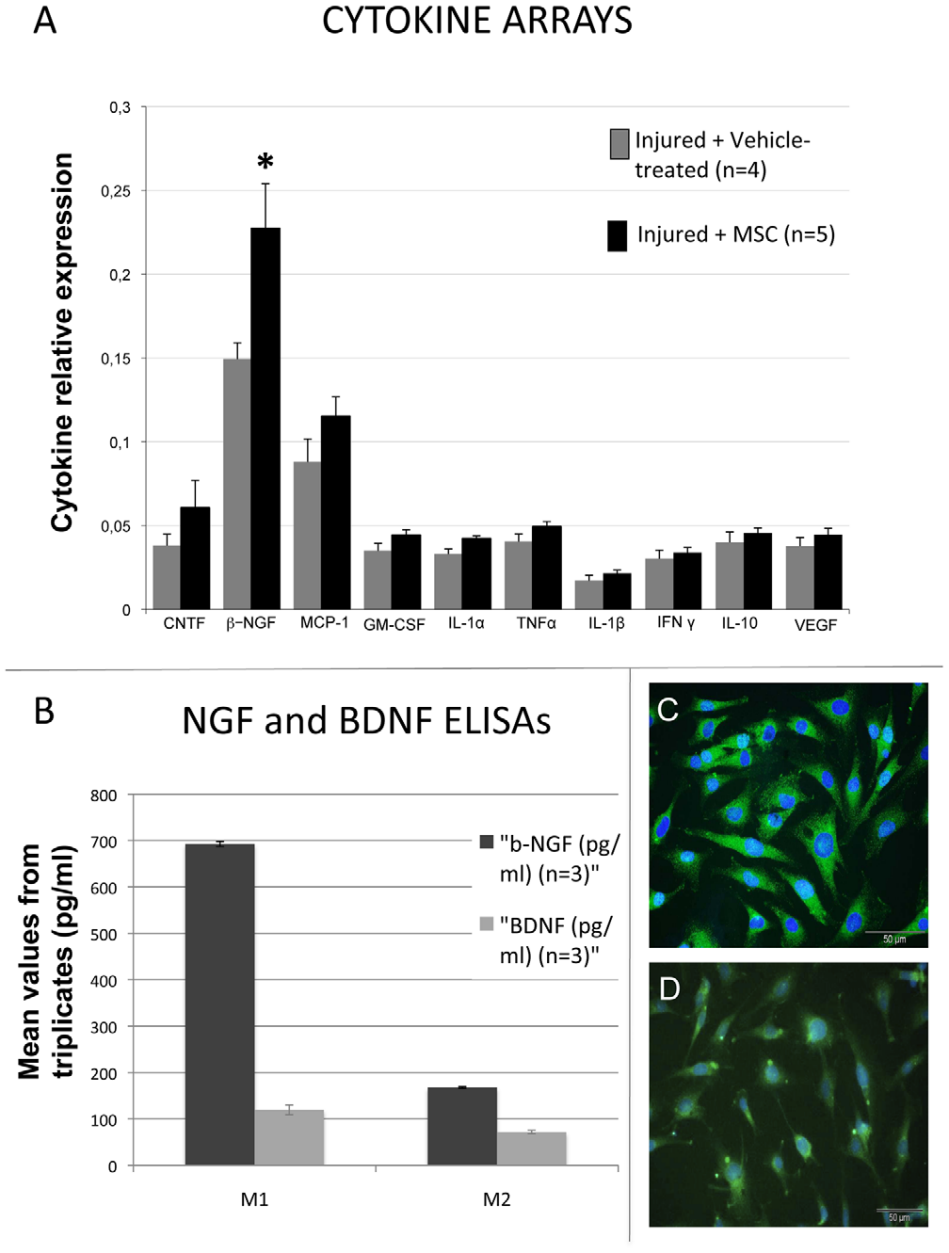
Cytokine array and Elisa results. (**A**) Cytokine arrays. Spinal cord extracts from injured vehicle-treated (n = 4) and MSC-treated (n = 5) rats. ß-NGF is significantly increased within the lesioned site 3 days after MSC injection compared to controls. * p<0.05 (mean±S.E.). (**B**) Histogram showing the amounts, in pg/ml, of the neurotrophins NGF and BDNF as quantified by Elisa, within 2 distinct P12 MSC-conditioned media (M1 and M2). (**C**) NGF and (**D**) BDNF fluorescent immunocytochemistry on P12 MSCs *in vitro*. Scale bar : 50 µm.

As behavioural improvements appear already during the first week following MSC graft, we performed cytokine array on proteins extracted from the injured spinal cord of control and MSC-treated rats (3 days post-graft), to assess and compare changes in cytokine/trophic factor expression. We analyzed the expression of Ciliary neurotrophic factor (CNTF), Granulocyte macrophage-colony stimulating factor (GM–CSF), Interleukin-1 alpha (IL–1α), Interleukin−1 beta (IL–1β), Vascular endothelial growth factor (VEGF), Nerve growth factor beta (ßNGF), Interleukin-10 (IL–10), Interferon-gamma (IFN-γ), Monocyte chemo-attractant protein-1 (MCP–1) and Tumor necrosis factor alpha (TNF–α). The expression levels of all these molecules were higher in grafted animals compared to vehicle-treated animals. The sole factor for which we observed a difference statistically significant is ßNGF (p = 0.042) (figure 5A). All other tested molecules showed only a slight increase tendency. As SCI is known to trigger NGF up-regulation by itself, the higher rate of NGF expression obtained 3 days after the graft in the “injured + MSC group” compared to the “injured + vehicle-treated group”, raises the hypothesis that the source of this increased NGF are the grafted MSCs. We thus checked if MSCs produce NGF by performing an ELISA assay on P12-MSC conditioned medium. The analysis of 2 different culture media (from 2 distinct cultures) demonstrates that MSCs secrete the neurotrophin NGF (figure 5B). Immunocytochemistry for NGF on P12 MSCs in culture also reveals that all of them express NGF *in vitro* (figure 5C). In parallel, ELISA for BDNF was performed on the same culture media, showing that MSC do also secrete BDNF, but in a much lower quantity compared to NGF (figure 5B). This correlates with the weaker BDNF expression obtained by immunocytochemistry on MSCs *in vitro* (figure 5D).

### 7. MSC graft promotes tissue sparing after spinal cord compression injury

**Figure 6:**
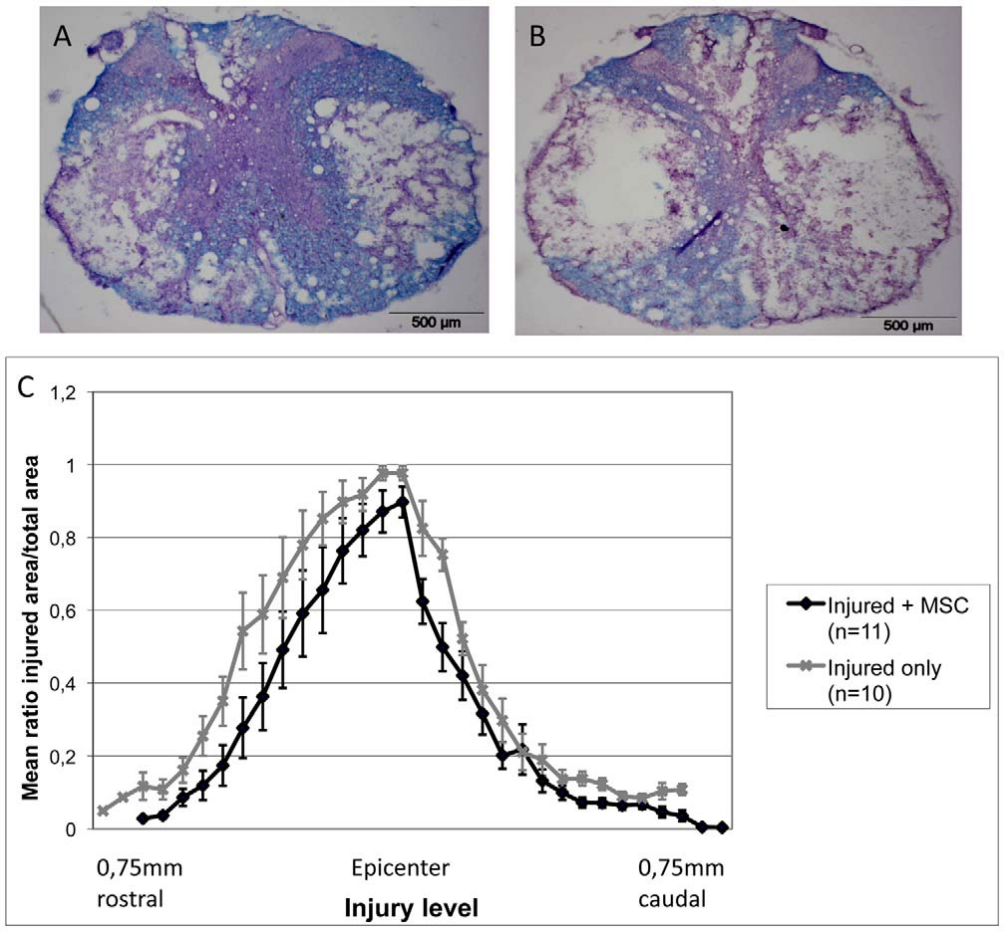
Neuroprotection. MSC transplants favor tissue sparing after SCI. Luxol fast blue/hematoxylin staining on cross sections of MSC-grafted (**A**) and control injured only (**B**) rats, 21 days after transplantation. The quantification of spared tissue (**C**), as assessed by the mean ratio of injured area on total area of the sections, reveals a significant decrease of the lesion extension in MSC treated rats compared to control ones. * p<0.05.

To further elucidate the mechanisms that could explain the beneficial effects obtained with MSC transplantation, we investigated the possibility of a tissue sparing effect. We thus performed a histological luxol fast blue/eosin staining on serial cross sections from injured spinal cord at 28 days after injury, in MSC grafted and in control “injured-only” rats. Sections were then analyzed and the ratio “injured area/total area” from each section determined. (figures 6A–B). The mean values were reported on figure 6C. This analysis revealed a reduced surface of damaged tissue for the grafted group compared to the control one (white matter and grey matter together). The difference was statistically significant (p = 0.0454). It thus strongly supports a neuroprotective effect of the MSC graft.

### 8. MSC graft does not increase axonal regrowth

**Figure 7:**
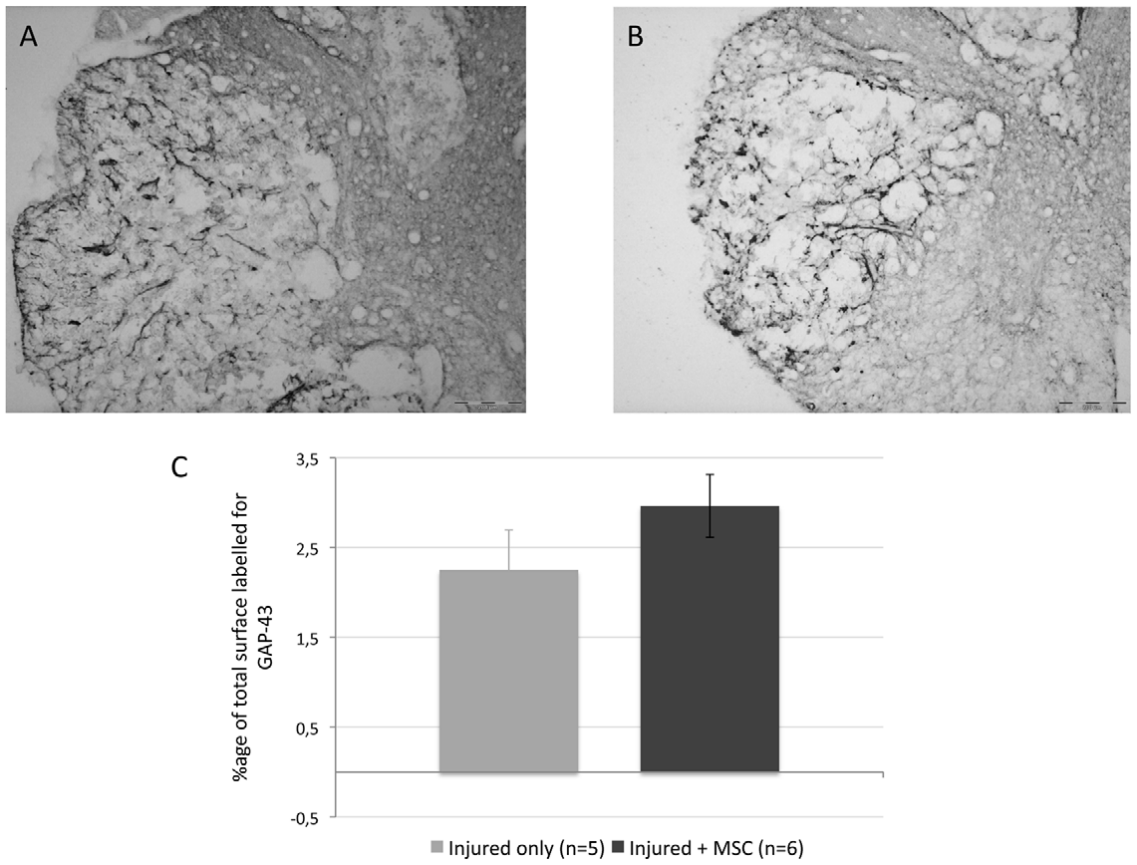
Axonal regrowth. (**A**–**B**) GAP43 immunoreactivity on transversal spinal cord sections, 28 days after SCI, in injured + MSC (A) and injured-only (B) groups. Sections were taken at the lesion site. (**C**) Image analysis doesn't show a significant difference (Student's t-Test, p = 0,23) in the percentage of total lesioned area immunoreactive for GAP43 between treated (2,96 ± 0,35 %) and control (2,25 ± 0,44 %) groups

We analysed the effect of MSC graft on axonal regrowth 21 days after transplantation, by an immunostaining against GAP-43 on transversal spinal cord sections taken at the level of the lesion site (figures 7A–B). The quantification of the proportion of the total surface of the section stained for GAP–43 doesn't show a significant difference between control (2,25%±0,44) and grafted (2,96%±0,35) animals, though these latter exhibit higher values of GAP-43 immunoreactivity (figure 7C; p = 0.23 Student's t-Test).

### 9. MSC graft favours vascularisation

**Figure 8:**
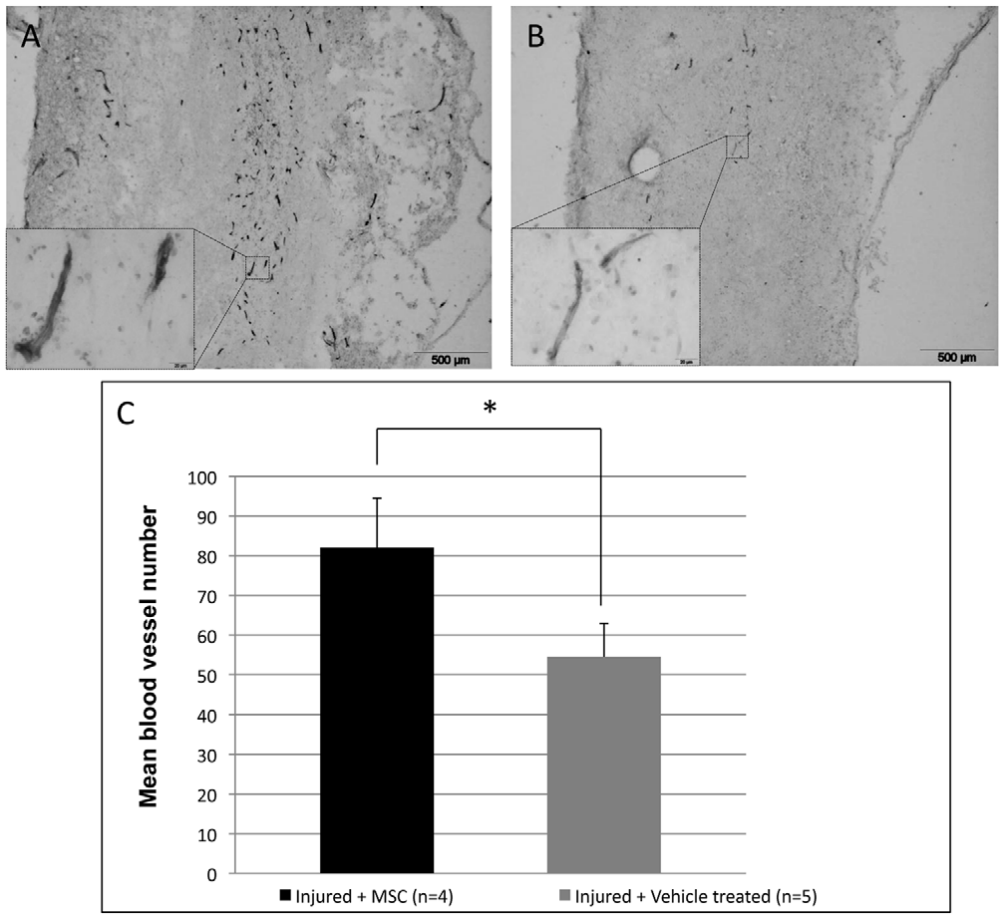
Blood vessel quantification. (**A**–**B**) RECA-1 immunohistological staining on longitudinal sections of MSC-treated (**A**) and control vehicle-treated (**B**) spinal cords. Scale bar: 500 µm. (**C**) Blood vessel quantification within the lesioned site reveals a significant increase in MSC treated rats compared to control-vehicle treated ones. *p<0.05.

To assess a potential effect of MSC graft on tissue vascularisation, we performed a RECA-1 immunostaining, a specific marker for rat endothelial cells, on longitudinal spinal cord sections centred on the lesion, both on MSC-treated and control groups (3 days after MSC or vehicle treatment). A first qualitative analysis revealed a higher number of blood vessels in the injury epicentre of MSC-treated cords compared to controls (figures 8 A–B). Quantification confirmed a significant increase in the number of blood vessels in the injury site of grafted animals compared to controls (figure 8C). Despite the higher surface analysed in the control group (which correspond to the injured tissue: 2.067.378±198.630 µm^2^ for injured + MSC and 2.207.101± 212?647 µm^2^ for vehicle-treated), the number of blood vessels is lower in that group compared to injured + MSC group.

## Discussion

Our work confirms that MSC treatment is beneficial after SCI and is in accordance with previous studies reviewed by [4]. We show by two complementary locomotor tests that MSCs have the ability to significantly improve functional recovery. Using the open field test with the BBB rating scale, we were able to precisely study locomotion faculties after SCI. The animals grafted with MSCs do not only reach higher motor scores, but were also able to support their weight and make coordinated steps whereas control animals were not able of weight support and seemed to reach a plateau. MSCs appear to act quickly after iv injection as the difference of recovery of MSC-grafted animals and control groups is already important 3 days after the cell injection (BBB scores of 4.5 and 2.6 respectively). We also show a beneficial effect of MSCs on the grid-walking task. Again, MSC-grafted rats obtained higher scores than controls, reinforcing the conclusion that MSCs do really exert a beneficial effect on recovery after SCI. According to the literature [37], the evidence of a functional improvement after iv injection of MSCs in SCI is scarcely described. Indeed, our results can be compared to one single study, conducted by Urdzikova et al, who also described locomotor improvement after bone marrow stromal cell iv injection performed one week after a thoracic compression injury [38]. Their behavioral data also demonstrate a rapid effect of the treatment, but no evidence of MSC integration within the host tissue to replace lost cells has been described, even if some grafted MSCs could be found within the lesioned tissue.

In spite of the efficacy and maintenance of the BrdU labeling of MSCs, the use of an effective protocol to detect BrdU *in vivo*, and the confirmation of the viability of the cells at the time of injection, we were not able to find the grafted MSCs at various delays (7, 14 and 21 days post injection) in the injured spinal cord, neither in highly vascularized organs such as lungs, liver, spleen and kidneys where we thought the cells could have migrate after systemic injection [39], [40]. These observations are in accordance with Karp and Teo analysis of exogenously infused-MSC homing [41]. Indeed, according to this review, the homing of cultured-expanded iv-injected MSCs is inefficient, and apparently due to (i) a lack of relevant cell adhesion and chemokine receptors and (ii) to the increased size of MSCs in culture, which likely promotes cell entrapment and reduces the number of MSCs that reach the target site. If homing towards the injury site of MSCs delivered by iv injections has nevertheless been reported in SCI [13], [38], [42] as well as in brain injuries [40], [43], these cells reach the host tissue in small numbers and are not sufficient to explain the rapid functional recovery [44]. It thus seems clear that iv delivery of MSCs is not the most effective way to introduce cells after injury (see also [45], [46]) but it nevertheless remains the least invasive, making it worth trying. Other delivery methods (intra-thecal, intra-arterial) might have better potential in the perspective of clinical application [47]. Yet, it remains that even if we couldn't find grafted cells in the host tissue, we observed a clear, significant and early beneficial effect of MSC iv transplantation on locomotion, strongly suggesting that MSCs act throughout their secretions. Likewise, several reviews on the paracrine therapeutic effect of MSCs [4], [20], [21], [23] reinforce our findings.

To explore this hypothesis of an early paracrine effect of the MSCs, we performed cytokine array on the injured tissue 3 days post injection. Among the cytokines analyzed, we observed a slight increase in the expression of CNTF, MCP-1 and GM-CSF. CNTF plays a role in neuroprotection [48] and promotes survival and differentiation of oligodendrocyte precursor cells (OPCs) after SCI [49]. MCP-1 induces monocyte recruitment during inflammation [50], enhancing myelin debris clearance in CNS injuries [51]. GM-CSF activates macrophages [51], inhibits apoptosis of neuronal cells and gliosis after SCI [52], [53] and treatment with GM-CSF or GM-CSF with bone marrow stromal cell transplantation promotes axonal regeneration and functional recovery after SCI [54], [55]. Several authors reviewed the therapeutic properties of MSCs and reported that these cells are able to secrete, among others, CNTF, MCP-1 and GM-CSF [21], [22], [23]. We also detected a slight tendency of increased expression of anti-inflammatory cytokines IFN-γ, IL-10, and pro-inflammatory IL-1β after MSC injection but the levels concerned and the differences with the control group are insignificant. All together these cytokines and trophic factors could have a beneficial effect after SCI on the reduction of the inflammatory reaction, cell death and tissue protection, but regarding the low difference we have seen in their expression in injured tissue between MSC-grafted and control rats, they cannot be the only explanation for an important functional recovery.

Concerning nerve growth factor (NGF), we observed a high and statistically significant increase of its expression in MSC-grafted animals compared to controls, which totally agrees with the results obtained after MSC iv transplantation in traumatic brain injury [56]. NGF has been shown to play a role in protecting neuronal injured tissue from toxic events, inducing regrowth, repair and reorganization of neuronal connections [57], [58], [59] but also stimulating neurogenesis [22], [60]. NGF is also known to activate monocytes, macrophages and microglia in CNS by increasing their phagocytic activity [57], [61], [62]. After CNS injury, NGF is secreted by microglia [63], but we believe that the higher NGF expression observed in the injured tissue in MSC-grafted spinal cord results from its secretion by MSC themselves. Indeed, we show that they do express NGF *in vitro*, and that they secrete NGF as demonstrated by our Elisa results. BDNF, to a lesser extent, is also expressed and secreted by MSCs *in vitro*. These results are in accordance with the literature [22], [64], [65]. We however can not rule out the possibility that the elevated expression of NGF found in the grafted tissue could be due to endogenous cells being stimulated by injected MSCs. BDNF is beneficial to SCI repair, both for axonal growth and tissue protection [66], [67]. If no significant effect on axonal regrowth with GAP-43 staining was obtained in our MSC-treated rats, we however show a neuroprotective effect of our transplanted cells. Actually, one of the most documented effects of MSC treatment after SCI is the reduction of cavity formation or tissue sparing, which is frequently reported without the demonstration of an axonal regrowth-associated effect [45], [68], [69], [70], [71]. Bearing in mind the large neuroprotective and repair-inducing effect of NGF, its higher expression could thus partially explain the largest amount of spared tissue in MSC grafted rats.

Among the molecules secreted by MSCs, pro-angiogenic factors like VEGF [72] are interesting to study in SCI as blood supply is essential for repair of damaged tissue. Furthermore, tissue sparing is often associated with stimulation of angiogenesis. VEGF/PDGF-stimulated angiogenesis results in lesion size reduction and in white matter sparing with functional outcome after SCI [73], [74]. In our study, we only detect a slight tendency of increase in the expression of VEGF after MSC graft, which is probably not sufficient to be responsible of the higher blood vessel density that we found. As it has been previously shown, MSCs have the capacity to provide other trophic support for angiogenesis such as bFGF, BMP-2, angiogenin, MCP-1 or IL-6 [72], [75]. Matrix metalloproteinases also facilitate angiogenesis by helping the invasion of endothelial cells [76], [77]. MSCs could also be able to increase endothelial cell survival in an injured environment via other paracrine secretions like anti-apoptotic molecules [20], [21], [23].

Throughout the literature, MSCs show differences in their survival and integration within host tissue as well as in their effects on repair. We explain these discrepancies by the difference between MSCs themselves. Indeed, variations in the morphology, behavior and characteristics have been shown depending on the number of passages, the plating densities and the duration of culture [78]. It has been reported that subpopulations of MSCs with different properties do exist [64], [79]. Using different markers, some authors were able to select subpopulations of MSCs [80]. Furthermore, it has been shown that MSCs in the bone marrow have at least two developmental origins, one of them being the neural crest [81], [82]. Also, it has been reported that deprivation of serum [83], [84] or hypoxia culture conditions [85] modify MSC characteristics. The existence of such subpopulations with distinctive properties is reinforced by differences in MSC properties in culture or in their post-graft effect, even more for human MSC from different donors [86], [87]. So, we believe the way a heterogeneous population of MSCs is cultured selects some subpopulations with different characteristics and properties.

In conclusion, despite differences in the mechanisms and effects of MSC graft after SCI, their beneficial effect on functional recovery remains constant. We confirm an early significant behavioral improvement in MSC-transplanted rats, which is likely due to their paracrine secretions, as no cells could be found within the host. We also show a tissue sparing effect and a higher blood vessel density in the injured site, which can also play a beneficial role in tissue sparing and functional recovery by improving blood flow in the lesion [88]. These effects are mediated at least partially through the important NGF secretion, but, in regard of the large array of factors MSCs are able to secrete, we cannot exclude the effect of other molecular actors we didn't study here. Additional work is needed to study and further characterize the paracrine effect of MSCs after SCI.

## Materials and Methods

### 1. Mesenchymal stem cell isolation, culture and characterization

MSCs were harvested from Wistar adult female rats. After sacrifice, femurs and tibias were aseptically removed and bone marrow flushed from each piece with approximately 5 ml culture medium using a sterile syringe. Bone marrow was placed in T75 culture flask in Dulbecco's Modified Eagle Medium (Invitrogen) supplemented with 10% of foetal bovine serum (Invitrogen). The culture medium was changed once per day for three days in order to remove non-adherent cells. Then the culture medium was changed twice a week and cells were passaged when they reached confluence. The enrichment in MSCs was made simply by several passages due to their preferential adherence to plastic. Cells were characterized by their specific surface antigen expression with immunocytochemistry against CD90 (+), CD271 ( =  p75NGFr) (+) [34], CD11b (−) and CD45 (−). Their multipotency was monitored by *in vitro* differentiation into adipocytes and osteocytes [35], [84]. After fixation with 4% paraformaldehyde, the adipogenic differentiation was evaluated by accumulation of lipid vacuoles and staining with Oil Red O (Sigma) and the osteogenic differentiation was visualized by Alizarin Red S staining (Sigma) of matrix mineralization associated with osteoblasts. We considered that the culture contained 95% of MSCs after 4 to 5 passages.

### 2. Animals

50 adult female Wistar rats from the animal facility of the University of Liege were used for this study (±250 g). All experiments were conducted according to the ethical laws and regulations of Belgian National Fund for Scientific Research. The project research has been approved by the ethical commission for animal use of the University of Liege, Belgium. Animals were subdivided into 3 experimental groups (Table 1):

**Table 1:** Animal distribution in the various experimental groups.

	Injured + MSC -treated	Injured + Vehicle -treated	Injured only
4 weeks survival after SCI	N = 11	N = 11	N = 10
10 days survival after SCI	N = 9	N = 9	/

1. Spinal cord injured rats without treatment (n = 10)  =  Injured only.
2. Spinal cord injured rats injected with fresh culture medium without serum (vehicle) (n = 20)  =  Injured + vehicle-treated.
3. Spinal cord injured rats injected with a suspension of MSCs in fresh culture medium without serum (n = 20)  =  Injured +MSC-treated.

Animals were housed individually with water and food *ad libitum*. One week before the beginning of the experiments, animals were placed daily in the different surroundings of the behavioural tests, to get accustomed to them.

### 3. Spinal cord injury procedure and animal care

Animals were anaesthetized by inhalation of Isoflurane (Forene®, Abbott, Queenborough, Kent, England) 2 to 3% in a flow of 1 L/minute of oxygen. The compression injury was performed as described by Vanicky et al. [89]. Briefly, a 2-French Fogarty arterial embolectomy catheter (Edwards Lifesciences LLC, Irvine, CA, USA) was inserted in the epidural space at the T10 level and moved rostrally for 2 metameric levels before being inflated with a distilled water volume of 15 µl and left in place during 5 minutes. The balloon was then deflated and carefully removed. Skin and muscle were carefully closed in two layers. 1mL of NaCl 0.09% was injected subcutaneously to prevent dehydration during the time animals recover from anaesthesia. Antibiotic (amoxicilline-clavulanic acid) was administrated by intraperitoneal (ip) injection after the surgery to prevent urinary tract infection. Animals had their bladder manually emptied twice daily until recovery of micturition and were carefully inspected for weight loss, dehydration and autophagia.

### 4. Mesenchymal stem cell injection procedure

72 hours before injection, MSCs were cultured in D-MEM (Invitrogen) without serum to induce the expression of nestin, a protein expressed by neural stem cells. This was performed in order to favour their fate towards neural stem cells [84], which could further support their integration within the host tissue. In order to label MSCs, 1.10^−6^M of 5-Bromo-2′-deoxyuridine (BrdU) was added to the culture medium during this period. The culture medium was changed every 24 hours. MSCs were injected 7 days after injury. We used passage 12 MSCs. After reaching confluence, cells were detached and collected in D-MEM without serum. The number of cells per mL was calculated and cells were diluted in order to obtain 1.10^6^ cells in an injection volume of 500 µL. Animals were anaesthetized by inhalation of Isoflurane (Forene®, Abbott, Queenborough, Kent, England) 2 to 3% in a flow of 1 L/minute of oxygen. Intravenous 24 GA catheter (Becton Dickinson) was inserted in one of the caudal vein and the cell suspension was injected with the UltraMicroPump (UMP-3) and SYS-Micro4 controller (World Precision Instruments) in a flow rate of 50 µL/min. The catheter was removed 2 min after the end of injection to avoid cell loss during its withdrawal. The last drop of cell suspension collected from the catheter was put back in culture on polyornithine-coated glass coverslips to assess cell viability at the time of the injection procedure.

### 5. Behavioural analyses

Following SCI, locomotion and sensorimotor skills were monitored as follows, twice a week, during 3 weeks:

#### Open field locomotor test

The Basso, Beattie and Bresnahan (BBB) locomotor rating scale [90] was used. Rats were observed walking freely in an open field and scores were attributed by two blinded observers according to the recovery of hind limb movements.

#### Grid navigation test

This test was used to assess the deficit in descending motor control. The animals had to cross 5 times a 1 m long grid with round metal bars separated from 4 cm and a score was attributed by two blinded observers according to the recovery of hind limb movements: 0. Hindlimb drag, no foot placement; 1. Consistent foot faults, foot miss and fall through wire mesh; 2. Consistent foot faults, foot slip off grid after accurate step and fall through wire mesh; 3. Accurate foot placement on grid but toes do not grip; 4. Normal course over grid with gripping toes.

For statistical analysis, we used a GLMM model (generalized linear mixed models) and p<0.05 was considered statistically significant.

### 6. Histological analyses

#### Tissue processing

Animals were deeply anaesthetized with pentobarbital (200 mg/kg) ip injection (Nembutal, CEVA Santé Animale, Libourne, France) and perfused with 250 mL of NaCl 0.09% (4°C) followed by 500 mL of 4% paraformaldehyde (4°C) in 0.1 M phosphate buffer saline (PBS, pH 7.4). The spinal cords were rapidly dissected out and post-fixed in the same fixative for 24 h at 4°C and then kept for 48 h in 30% sucrose for cryoprotection. Two tissue blocks of 0.75 cm length were taken from both sides of the lesion epicentre. Tissue blocks were then either transversely, either longitudinally, cryo-sectioned in 20 µm serial sections, mounted on superfrost^+^ glass slides and stored at −20°C until used.

#### Tissue staining

To analyze the lesion extension, we performed Eosin – Luxol fast blue histological staining. We calculated the ratio between the measured injured area and the entire area of the spinal cord slice on serial transversal sections, covering a tissue bloc of 1,5 cm centered on the lesion epicenter. We used ANOVA followed by the Scheffé's post-hoc test. p<0.05 was considered statistically significant.

#### BrdU immunostainings

Sections were fixed with 4% PFA for 5 min. After 1 TBS (Tris buffered saline) rinse, tissue sections were first permeabilized with 0.3% Triton in TBS for 15 min at RT. They were then rinsed 3×5 min in TBS at 37°C, and further incubated in HCl 2N for 30 min at 37°C followed by Na Tetraborate 0.1 M pH 8.5 for 10 min at 37°C. After 3 TBS rinses, they were incubated in a blocking buffer (1% BSA and 10% goat normal serum in 0.3% Triton X-100-TBS) for 1h, and thereafter overnight at 4°C in the BrdU antibody diluted in the blocking buffer (containing 3% of normal goat serum instead of 10%). After 3 TBS−0.05% Tween20 and one TBS rinses, the sections were incubated for 1 h at RT with Alexa Fluor®488 goat anti-rat (1/1000, Invitrogen), rinsed 3 times in TBS−0.05%Tween20, and once in TBS. Sections were finally coverslipped with Vectashield® with DAPI mounting medium (Labconsult). Negative controls were obtained by omission of the primary antibody.

In order to check the maintenance of BrdU within MSCs throughout their divisions, we performed a BrdU immunocytochemistry on P12 MSCs as follows: P12-MSCs were cultured in D-MEM +10% FBS until 60% confluence. Cells were then rinsed once with PBS and cultured in D-MEM without FBS + 1.10^−6^ M BrdU for 3 days (as it was done for the cells used for *in vivo* transplantation). The medium was renewed every day. Then, MSCs were placed again in D-MEM +10% FBS for 3, 7 and 21 days. During this period, the culture was maintained by medium changes and passages (0, 1 and 6 passages, respectively). At each delay, cells were detached and placed on glass coverslips during a few hours in order to perform a BrdU immunostaining.

#### Fluorescent immunocytochemistry

Cells were cultured on polyornithine (Sigma-Aldrich, Schnelldorf, Germany) coated glass coverslips and fixed during 5 minutes in 4% PFA or in 4% PFA buffered in 50 mM sodium borate at pH 9.5 for Nestin immunostaining. For BrdU staining, cells were fixed with 4% PFA for 5 min, incubated in HCl 2N at 37°C for 30 min and neutralized in sodium tetraborate 0,1 M for 10 min. After 3 PBS rinses, cells were incubated in a blocking buffer (1% BSA and 10% of appropriate normal serum in PBS +0.3% Triton X-100) for 1 h, and thereafter overnight at RT in the primary antibody diluted in the blocking buffer (1% BSA and 3% appropriate normal serum in PBS +0.3% Triton X-100). After 3 PBS−0.05% Tween20 and one PBS rinses, cells were incubated for 1 h at RT with the secondary antibody diluted in PBS +1% of appropriate normal serum, rinsed 3 times in PBS +0.05% Tween20, and once in PBS. Coverslips were finally turned over on glass slide using Vectashield® with DAPI mounting medium (Labconsult). Negative controls were obtained by omission of the primary antibody.

#### DAB immunostainings

Tissues were fixed with 4% PFA for 5 min, then incubated in a 0.3% H_2_O_2_, 0.1% Na azide solution in PBS for 20 min at RT to reduce endogenous peroxydase activity. Nonspecific binding was prevented by 1h incubation in 3% normal serum and 1% bovine serum albumin solutions in PBS +0.2% triton. Sections were then incubated overnight at RT with the primary antibody. After 3 PBS rinses, they were incubated with their respective secondary biotinylated antibodies (Vector Laboratories) diluted and centrifuged in PBS with 1% BSA and 2% of rat serum, for 1 h at RT. Then sections were incubated 1 h with the avidin-biotin-peroxydase complex (Vector Laboratories), diluted 1/1000 in PBS and the immunostaining revealed with 3,3′-diaminobenzidine.

#### Antibodies

The primary antibodies used for immunostaining were the following: mouse anti-CD90 (1/100, AbCam), rabbit anti-CD45 (1/100, AbCam), mouse anti-CD11b (1/200, AbD Serotec), mouse anti-Nestin (1/200, Chemicon), rat anti-BrdU (1/100, AbD serotec), rabbit anti-NGF (1/100, AbCam), rabbit anti-p75NGFr (1/500, AbCam), mouse anti-RECA-1 (1/200, AbCam), chicken anti-BDNF (1/250, R&D Systems). Secondary antibodies were rhodamine- or FITC-coupled donkey anti-mouse, rhodamine- or FITC- donkey anti-rabbit, rhodamine donkey anti-chicken (1/500, Jackson Immunoresearch), Alexa Fluor®488 goat anti-rat (1/1000, Invitrogen) or Alexa Fluor®488 donkey anti-mouse and anti-rabbit (1/500, Invitrogen) for immunofluorescence, and biotinylated goat anti-rabbit and horse anti-mouse (1/500, Vector Laboratories) for DAB immunostainings.

#### Image analysis

Immunoreactivity and tissue stainings were examined and captured using an Olympus AX70 microscope equipped with an Olympus DP50 digital camera and an Olympus FSX100 microscope. Images were analysed by AnalySIS software (Soft Imaging System, Olympus) for spared tissue quantification. For GAP-43 immunostaining quantification, bright field images of transversal sections of the “lesion-caudal” tissue block were taken and pictures were converted to gray scale. A threshold intensity of gray-colored staining was fixed for each slice. Then, a surface covering the entire lesion area was selected ( =  total lesion area). Thereafter, immunostaining was quantified within non-overlapping successive fields covering the total lesion area ( =  total stained area) using Analysis software (Olympus). Data were expressed as the percentage of total stained area (pixel) per total lesioned area (pixel). The results represent averages from 5 “injured only” rats and 6 “injured + MSC” rats. The statistical significances between injured-grafted and injured only groups were assessed using a Student t-test.

#### Blood vessel quantification

We performed RECA-1 immunostainings on longitudinal sections of spinal cords 10 days after injury (3 days after MSC graft for treated animals). The epicentre zone was encircled and the number of blood vessels counted within the zone. The statistical significances between injured-grafted and injured only groups were assessed using a Student t-test.

### 7. Cytokine array

We used a custom Rat Cytokine Antibody Array kit (RayBiotech, Norcross, Georgia, USA). Animals were deeply anaesthetized with pentobarbital (200 mg/kg) ip injection and perfused with 250 mL NaCl 0,09% at 4°C. Tissue block of 1 cm length and centred on the injury epicentre was dissected out and crushed into lysis buffer (RayBiotech) containing protease inhibitors (complete mini, Roche) on ice. After protein dosage (BCA protein assay reagent kit, Pierce), 200 µg of total proteins were used per sample. Analysis was performed as recommended in the protocol supplied with the kit. The cytokine array membranes were scanned with a Las-4000 luminescent image analyser (Fujifilm, Tokyo, Japan). The intensity of the signal was measured with the Quantity One 1-D Analysis software (Bio-RAD Laboratories, Hercules, CA). The results were expressed as the mean relative signal intensity, i.e. the ratio of sample signal intensity/positive control signal intensity. Each signal intensity of positive controls and samples were normalized by background substraction. The statistical significances between injured-grafted and injured only groups were tested using a Student t-test.

### 8. NGF – BDNF Quantification in the MSC-conditioned medium

NGF and BDNF levels were measured by enzyme-linked immunosorbent assay according to the manufacturer's protocol (ChemiKine Nerve Growth Factor Sandwich ELISA kit CYT304 and ChemiKine Brain Derived Neurotrophic Factor, Sandwich ELISA kit CYT306, Chemicon). The conditioned media from 2 distinct cultures of rat MSCs at passage 12 were harvested and concentrated using Amicon Ultra-15 centrifugal filter units (Millipore). 100 µl of these two concentrated media (M1 and M2) were used for the assay.
